# Amygdalin potentiates the anti-cancer effect of Sorafenib on Ehrlich ascites carcinoma and ameliorates the associated liver damage

**DOI:** 10.1038/s41598-022-10517-0

**Published:** 2022-04-20

**Authors:** Attia Ahmed Attia, Afrah Fatthi Salama, Jayda G. Eldiasty, Sahar Abd El-Razik Mosallam, Sabry Ali El-Naggar, Mohammed Abu El-Magd, Hebatala M. Nasser, Alaa Elmetwalli

**Affiliations:** 1 Botany and Microbiology Department, Faculty of Science, Benha University, Benha, Egypt; 2 Biochemistry Department, Faculty of Science, Tanta University, Tanta, Egypt; 3 Biology Department, University College of Haqel, University of Tabuk, Tabuk, Saudi Arabia; 4 Zoology Department, Women’s College for Arts, Science and Education, Ain Shams University, Cairo, Egypt; 5 Physiology Department, Faculty of Science, Tanta University, Tanta, Egypt; 6 Department of Anatomy, Faculty of Veterinary Medicine, Kafrelsheikh University, Kafrelsheikh, Egypt; 7 Department of Clinical Trial Research Unit and Drug Discovery, Egyptian Liver Research Institute and Hospital (ELRIAH), Mansoura, Egypt

**Keywords:** Biochemistry, Drug discovery

## Abstract

The burden of cancer diseases is increasing every year, therefore, the demands to figure out novel drugs that can retain antitumor properties have been raised. This study aimed to investigate the anti-tumor properties of amygdalin (Amy) against Ehrlich ascites carcinoma (EAC) bearing mice and its protective properties against liver damage. Amy and the standard anticancer drug Sorafenib (Sor) were given alone or in combination to Swiss albino female mice that had been injected with EAC cells. Biochemical parameters of liver function (AST, ALT, GGT, total protein, albumin), tumor volume, oxidative stress [malondialdehyde, (MDA)] and antioxidative [superoxide dismutase (SOD), and reduced glutathione (GSH)] markers were measured. The hepatic expression of the antioxidant-related gene [nuclear factor erythroid-2-related factor 2 (*Nrf2*)], the migration-related gene [matrix metalloprotease 9 (*MMP9*)], and the angiogenesis-related gene [vascular endothelial growth factor (*VEGF*)] were evaluated by qPCR. The results revealed that EAC-bearing mice treated with Amy and/or Sor showed a decrease in the tumor burden and hepatic damage as evidenced by (1) decreased tumor volume, number of viable tumor cells; (2) increased number of dead tumor cells; (3) restored the liver function parameters; (4) reduced hepatic MDA levels; (5) enhanced hepatic GSH and SOD levels; (6) upregulated expression of *Nrf2*; (7) downregulated expression of *MMP9* and *VEGF*, and (8) improved hepatic structure. Among all treatments, mice co-treated with Amy (orally) and Sor (intraperitoneally) showed the best effect. With these results, we concluded that the Amy improved the antitumor effect of Sor and had a protective role on liver damage induced by EAC in mice.

## Introduction

Cancer is characterized by an uncontrolled division of cells which can spread by direct growth into the tissues through invasion or by metastasis^1^. Ehrlich ascites carcinoma (EAC) induces a local inflammatory reaction with cumulative vascular permeability which results in strong edema, progressive ascitic fluid formation, and cellular migration, which are essential for tumor growth^2–4^. Cancer therapy involves surgery, chemotherapy, radiation, hormonal, and biological approaches. Struggles have been made to recognize natural and synthetic anticancer with antioxidant properties^5–7^. Alternatively, both anticancer and herbal extract with antioxidant properties could be given together during cancer treatment. Indeed, some trials have been conducted and the obtained results revealed the presence of a dual synergistic effect between synthetic antitumor and herbal agent^1,8–10^.

Amygdalin (Amy, Vit B12) has been recently accepted as one of the main sources of cancer chemoprevention drugs due to their diverse pharmacological properties^11,12^. Amygdalin is rich in anti-cancer compounds such as hydrocyanic acid in addition to benzaldehyde, which can induce an analgesic action^13^. Co-administration of a synthetic anti-tumor agent with amygdalin relieves hepatotoxicity and liver fibrosis^14^. Sorafenib (Sor) is a drug that has been shown to prolong overall survival in patients with advanced liver cancer. Sorafenib is an oral multi-kinase inhibitor exerting its effects via RAF/MEK/ERK pathway^15^, vascular endothelial growth factor receptor (VEGFR)^16^, and platelet-derived growth factor receptor beta (PDGFR-β) tyrosine kinases^17^. Little data are available in the literature regarding the combined effect of Amy and Sor against tumor burden and the liver damage induced by EAC. Therefore, this study was conducted to investigate this effect.

## Results

### Effect of amygdalin and/or sorafenib on body weight

Among all groups, only Cnt, Amy, EAC, Amy + SorIP, and EAC + Amy showed significantly (P ≤ 0.05) higher body weight as revealed by positive values of the body weight change with highest value in EAC followed by Cnt, then EAC + Amy, and finally Amy and Amy + SorIP group (Table 1). Moreover, EAC mice treated with Sor either IP (EAC + SorIP) or orally (EAC + SorOS) showed a significant decrease in body weight compared to EAC and control groups. The treatment with Amy alone showed either a significant decrease or insignificant increase in body weight when compared with EAC or control (Cnt) group, respectively. Dual treatment with Amy and Sor (OS or IP) resulted in a significant decrease in the body weight as compared with EAC-treated and control mice (Table 1).

**Table 1:** Mice initial body weights and change in body weight in different experimental groups.

Groups	Initial body weight (g)	Body weight change (g)
Cnt	20.25 ± 0.75	4.26 ± 0.33^b^
Amy	21.47 ± 0.67	2.75 ± 0.12^c^
SorIP	21.75 ± 0.85	**− **7.23 ± 0.41^f^
SorOS	20.75 ± 0.65	**− **8.28 ± 0.60^f^
Amy + SorIP	21.31 ± 0.71	2.25 ± 0.12^c^
Amy + SorOS	21.00 ± 0.40	**− **4.22 ± 0.45^e^
EAC	20.48 ± 0.63	7.53 ± 0.92^a^
EAC + Amy	21.63 ± 0.55	4.62 ± 0.46^b^
EAC + SorIP	21.26 ± 0.40	**− **3.75 ± 0.30^e^
EAC + SorOS	20.60 ± 0.85	**− **3.52 ± 0.31^e^
EAC + Amy + SorIP	21.50 ± 0.69	**− **0.24 ± 0.10^d^
EAC + Amy + SorOS	21.06 ± 0.41	**− **2.87 ± 0.12^e^

Data were presented as means ± SEM. Small (a–f) letters showed the marked change at P ≤ 0.05. The significant were expressed by dissimilar letters in the same column. *Cnt* Control, *Amy* Amygdalin, *Sor* Sorafenib, *EAC* Ehrlich ascetic carcinoma, *IP* Intraperitoneal, *OS* Per os (oral).

### Effect of amygdalin and/or sorafenib on tumor volume and EAC cell count

**Figure 1:**
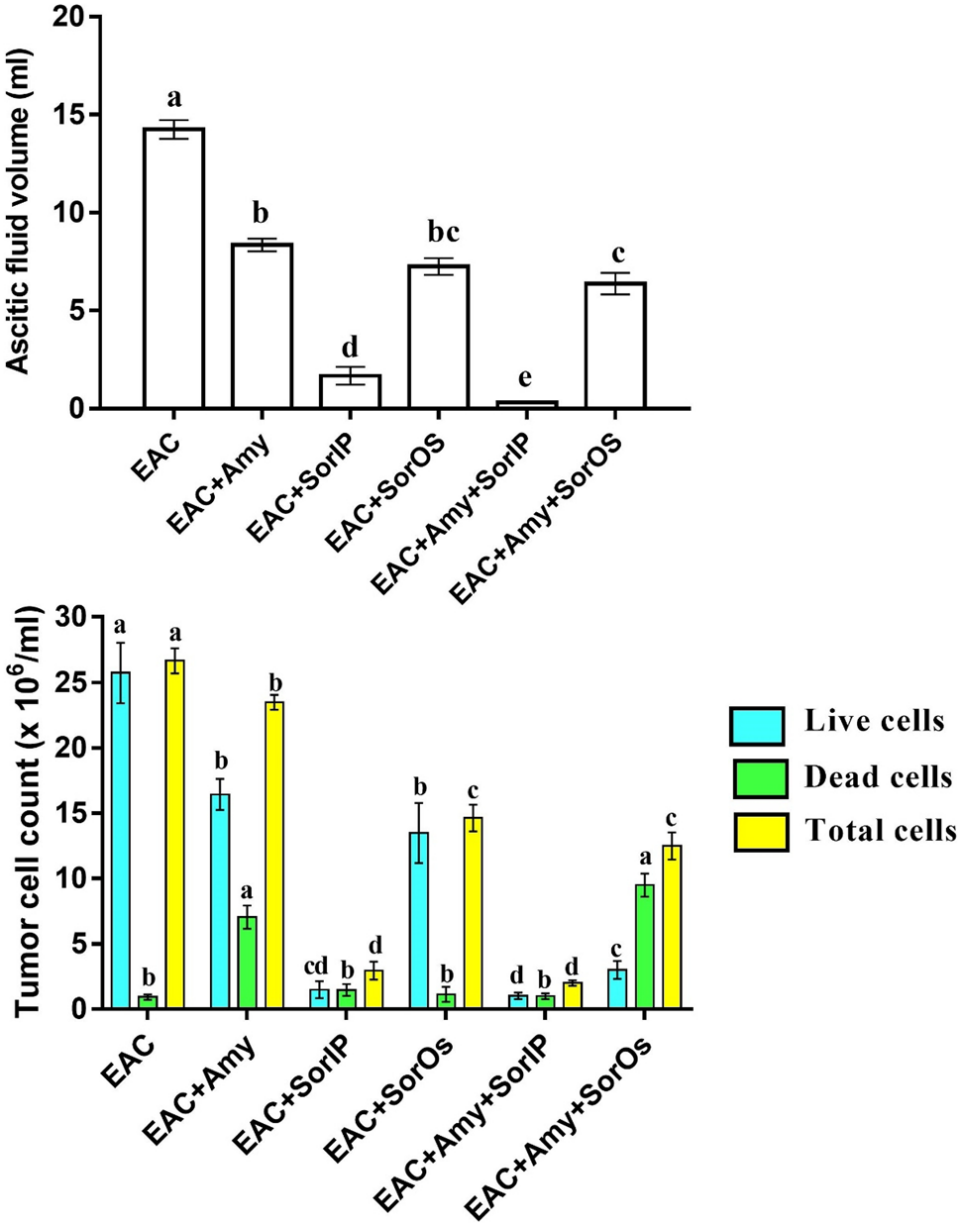
Tumor ascitic fluid volume and count of live and/or dead EAC cells in EAC-bearing mice after treatment with amygdalin and/or sorafenib. Data were presented as means ± SEM (n = 7). Small (a–e) letters showed the marked change at P ≤ 0.05. The significant were expressed by dissimilar letters above columns with the same color.

Tumor volume (volume of the ascitic fluid) in the EAC group was significantly (P < 0.05) higher than that in all other treated groups (Fig. 1). In the treated groups, EAC + Amy + SorIP group showed the lowest ascitic fluid volume followed by EAC + SorIP, then EAC + Amy + SorIP, EAC + SorOS, and finally EAC + Amy group. Treatment with Amy and Sor (IP or OS) alone or in combination significantly decreased the total and live tumor cells count, with lowest count in EAC + SorIP, and EAC + Amy + SorIP groups, followed by EAC + Amy + SorOS, EAC + SorOS, and finally EAC + Amy as compared to the EAC group (Fig. 1). However, dead cells were significantly higher in EAC + Amy and EAC + Amy + SorOS than other groups.

### Effect of amygdalin and/or sorafenib on liver functions

EAC mice treated with Amy and/or Sor showed significantly reduced serum levels of AST, ALT, and GGT, total protein, and a significantly higher level of albumin as compared to the untreated EAC mice (Table 2). The best improvement in these serum biochemical parameters was noticed in EAC + SorIP, and EAC + Amy + SorIP groups, followed by EAC + Amy + SorOS, EAC + SorOS, and finally EAC + Amy group. Additionally, serum ALT, AST, and GGT levels in SorIP, SorOS, Amy + SorIP, and Amy + SorOS groups were significantly increased compared to the control group. However, the Amy group showed insignificant change relative to the control group (Table 2).

**Table 2:** Liver function parameters in EAC-bearing mice after treatment with amygdalin and/or sorafenib.

Group	ALT (U/L)	AST (U/L)	Albumin (g/dL)	Total protein (g/dL)	GGT (U/L)
Cnt	44.75 ± 1.26^e,f^	59.50 ± 3.87^f^	4.34 ± 0.15^a^	6.12 ± 0.18^a^	19.75 ± 2.01^e^
Amy	48.25 ± 1.93^e^	62.75 ± 2.21^f^	3.67 ± 0.11^b^	5.15 ± 0.20^d^	22.00 ± 2.41^e^
SorIP	56.08 ± 1.58^d^	67.00 ± 2.73^e,f^	3.62 ± 0.16^b^	6.23 ± 0.22^a^	35.50 ± 2.75^c,d^
SorOS	54.21 ± 1.35^d^	69.21 ± 2.13^e^	3.40 ± 0.09^b^	5.37 ± 0.17^b,d^	33.75 ± 2.75^d^
Amy + SorIP	54.03 ± 1.84^d^	64.73 ± 2.28^a^	4.14 ± 0.13^a^	6.05 ± 0.19^a^	31.25 ± 3.25^d^
Amy + SorOS	57.50 ± 2.21^d^	77.50 ± 3.21^d^	3.62 ± 0.12^b^	5.50 ± 0.11^b^	38.00 ± 3.13^c^
EAC	86.47 ± 3.51^a^	165.0 ± 6.72^a^	2.35 ± 0.09^d^	4.17 ± 0.23^e^	154.37 ± 9.21^a^
EAC + Amy	75.61 ± 3.15^b^	103.8 ± 5.40^b^	3.13 ± 0.24^b^	5.65 ± 0.14^b^	99.50 ± 7.46^b^
EAC + SorIP	50.00 ± 2.38^d,e^	74.00 ± 3.80^d,e^	3.25 ± 0.11^b^	5.70 ± 0.16^b^	33.00 ± 2.13^d^
EAC + SorOS	66.50 ± 2.49^c^	88.00 ± 4.41^c^	3.07 ± 0.15^b^	5.97 ± 0.24^a,b^	46.25 ± 2.80^c^
EAC + Amy + SorIP	39.37 ± 1.48^f^	48.75 ± 2.15^g^	3.45 ± 0.13^b^	6.47 ± 0.17^a^	39.75 ± 2.13^c^
EAC + Amy + SorOS	50.25 ± 2.38^d,e^	75.75 ± 3.59^d^	3.20 ± 0.11^b^	5.73 ± 0.17^b^	34.30 ± 2.34^c,d^

Data were presented as means ± SEM. Small (a–e) letters showed the marked change at P ≤ 0.05. The significant were expressed by dissimilar letters in the same column.

### Effect of amygdalin and/or sorafenib on oxidative and antioxidative markers

Untreated EAC-bearing mice have significantly higher hepatic levels of lipid peroxidation marker MDA and lower hepatic levels of the antioxidant markers (GSH and SOD) than all control groups (Table 3). Treatment with Amy and/or Sor (IP and OS) restored these markers to levels near that of the control groups with best improvement (lowest MDA and highest GSH and SOD) observed in EAC + SorIP, and EAC + Amy + SorIP groups, followed by EAC + Amy + SorOS, EAC + SorOS, and finally EAC + Amy group.

**Table 3:** Hepatic oxidative (MDA) and antioxidative (GSH and SOD) parameters in EAC-bearing mice after treatment with amygdalin and/or sorafenib.

Groups	MDA (nmol/g tissue)	GSH (nmol/g tissue)	SOD (U/g tissue)
Cnt	3.98 ± 0.11^f^	6.31 ± 0.16^b^	58.63 ± 1.23^a^
Amy	4.01 ± 0.14^f^	7.22 ± 0.23^a^	54.09 ± 1.99^a^
SorIP	4.08 ± 0.09^f^	6.06 ± 0.19^b^	55.28 ± 1.76^a^
SorOS	4.17 ± 0.17^f^	6.13 ± 0.18^b^	56.21 ± 2.38^a^
Amy + SorIP	4.12 ± 0.15^f^	5.98 ± 0.14^b^	54.14 ± 2.14^a^
Amy + SorOS	4.23 ± 0.16^f^	6.04 ± 0.15^b^	56.82 ± 1.77^a^
EAC	23.58 ± 0.76^a^	3.23 ± 0.11^e^	25.26 ± 0.54^g^
EAC + Amy	15.37 ± 0.43^b^	4.43 ± 0.13^d^	32.12 ± 0.77^f^
EAC + SorIP	10.50 ± 0.35^d^	5.14 ± 0.14^c^	39.32 ± 0.54^d^
EAC + SorOS	13.51 ± 0.32^c^	5.03 ± 0.15^c^	36.39 ± 0.42^e^
EAC + Amy + SorIP	7.30 ± 0.26^e^	6.35 ± 0.22^b^	47.05 ± 1.01^b^
EAC + Amy + SorOS	9.76 ± 0.36^d^	5.33 ± 0.16^c^	42.31 ± 0.63^c^

Data were presented as means ± SEM. Small (a–g) letters showed the marked change at P ≤ 0.05. The significant were expressed by dissimilar letters in the same column.

### Effect of amygdalin and/or sorafenib on the expression of *Nrf2*,* MMP9*, and *VEGF* genes

**Figure 2:**
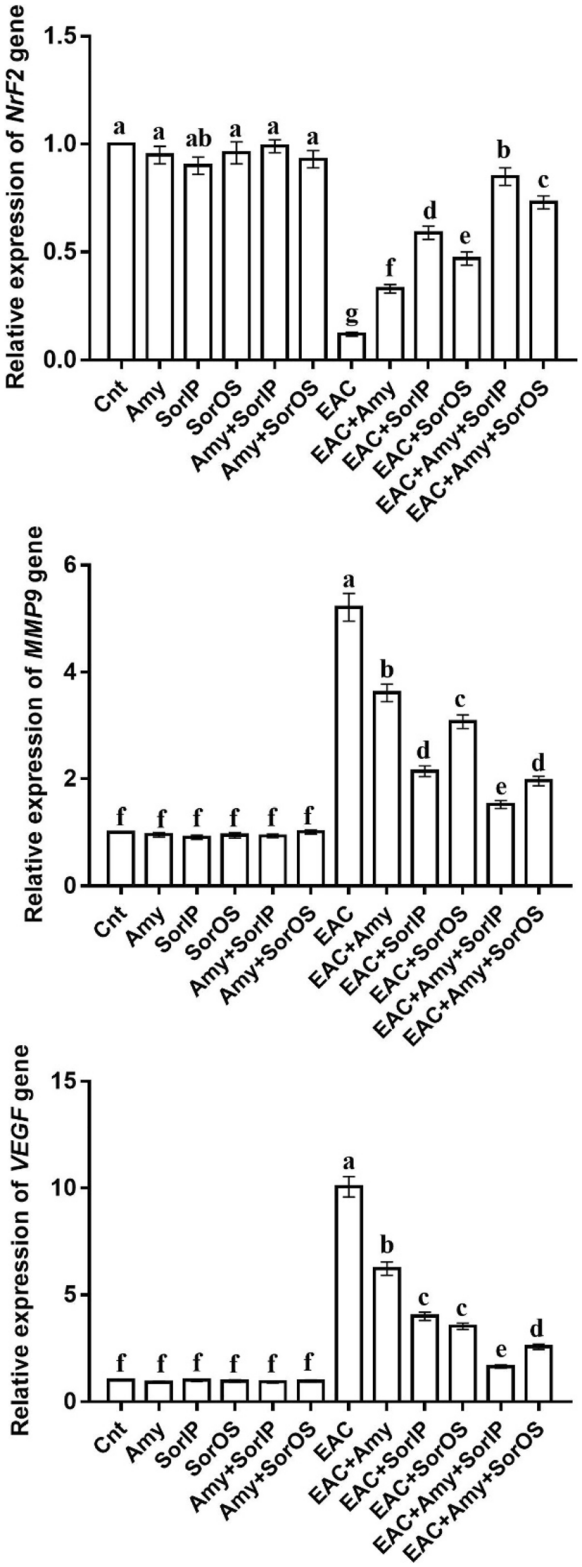
Changes in *Nrf2*, *MMP9*, and *VEGF* gene expression in liver of different groups as detected by real-time PCR. β-actin was used as internal control. The expression was expressed as fold changes mean ± SEM (n = 5/group). Columns with various letters [a (the highest fold change)–f (the lowest fold change)] showed significance at P < 0.05.

The obtained qPCR results revealed significantly downregulated hepatic expression of *Nrf2* and upregulated hepatic expression of *MMP9* and *VEGF* in the EAC group as compared to all control groups (Fig. 2). Administration of Amy and/or Sor (IP and OS) restored the expression of these genes to levels comparable to the control groups with best improvement (highest *Nrf2* and lowest *MMP9* and *VEGF*) noticed in EAC + Amy + SorIP, followed by EAC + Amy + SorOS, then EAC + SorIP, EAC + SorOS, and finally EAC + Amy group (Fig. 2).

### Histopathological examination

**Figure 3:**
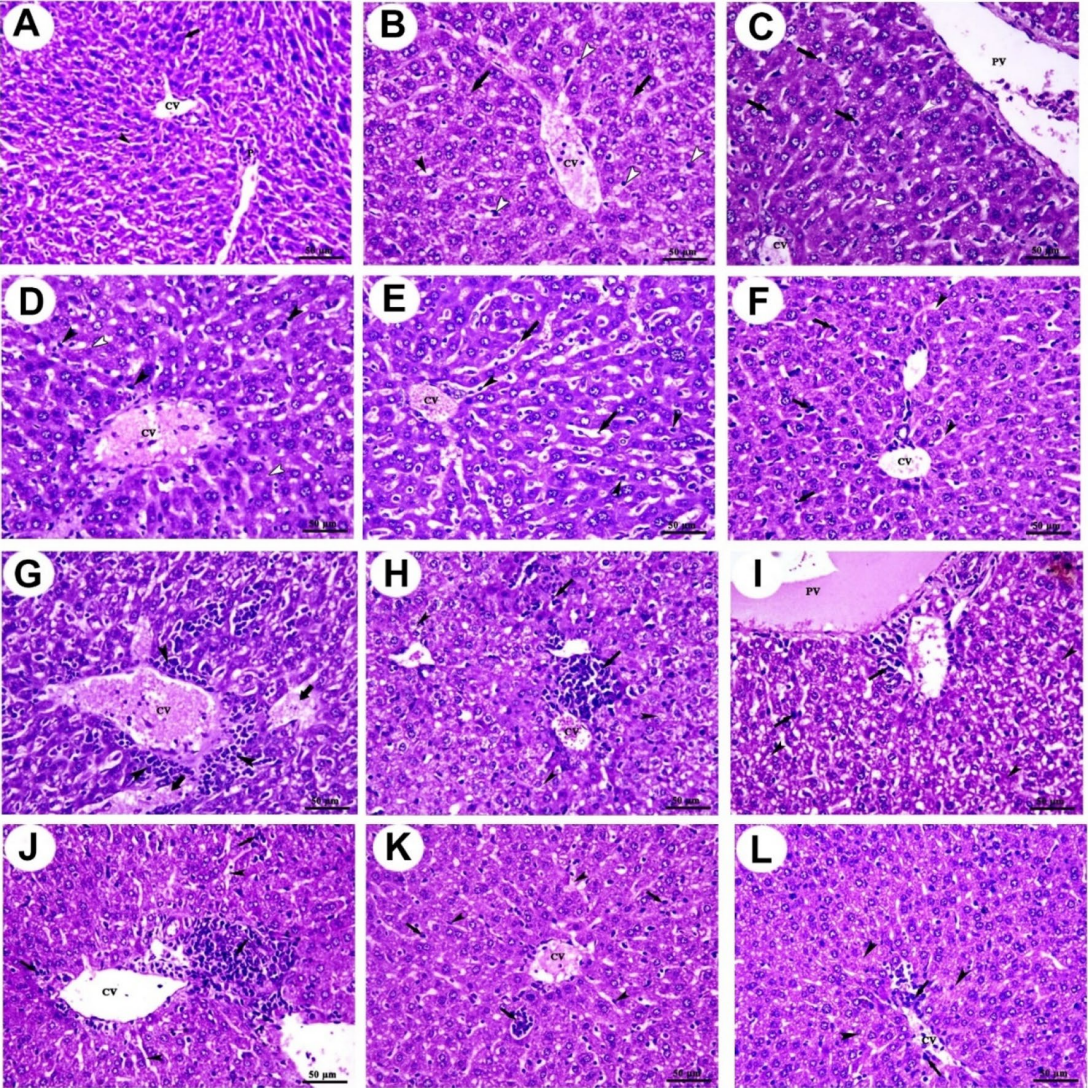
Photomicrographs of mice liver sections stained with H&E. (**A**) Control group shows central vein (CV), portal vein area (P), polyhedral-shaped hepatocytes (arrow), and blood sinusoids (arrowhead). (**B**) Amy group shows congested central vein (CV), mild dilation of blood sinusoids (black arrowhead), mild degeneration of hepatocytes (arrows), and mild Kupffer cells activity. (**C**) SorIP group shows congested central vein (CV), portal vein (PV), mild vacuolation of hepatocytes (white arrowheads), and moderate Kupffer cells activity (arrows). (**D**) SorOS group shows congested central vein (CV), mild vacuolation of hepatocytes (white arrowheads), and moderate Kupffer cells activity (black arrowheads). (**E**) Amy + SorIP group shows congested central vein (CV), congestion, and dilation of blood sinusoids (arrows) in addition to Kupffer cells activity (arrowheads). (**F**) Amy + SorOS group shows mild congestion of central vein (CV), mild dilation of blood sinusoids (arrowheads), and a moderate increase of Kupffer cells activity (arrows). (**G**) EAC group shows aggregations of pleomorphic, hyperchromatic, and darkly basophilic cells (arrowheads) around central vein (CV) and hepatocellular necrosis (arrows). (**H**) EAC + Amy group shows a moderate number of pleomorphic cells (arrows) with moderate degeneration (arrowheads). (**I**) EAC + SorIP group shows a small focal area of pleomorphic cells (arrows) with moderate degenerative changes (arrowheads). (**J**) EAC + SorOS group shows a moderate focal area of pleomorphic cells (arrows) and mild dilation of some blood sinusoids (arrowheads). (**K**) EAC + SorOS group shows the smallest focal area of the pleomorphic cells (arrows) with the mildest congestion in some blood sinusoids (arrowheads). (**L**) EAC + Amy + SorOS group shows small diffuse pleomorphic cells (arrows) with mild vacuolar degeneration of hepatocytes (arrowheads). Scale bars = 50 µm.

As shown in Fig. 3A, the liver of the control group showed normal hepatic architecture, central vein, portal vein area, polyhedral-shaped hepatocytes, and blood sinusoids. The liver of the Amy group showed congested central vein, mild dilation of blood sinusoids, mild degeneration of hepatocytes, and mild Kupffer cells activity (Fig. 3B). The liver of the SorIP group showed congested central vein, portal vein, mild vacuolation of hepatocytes, and moderate Kupffer cells activity (Fig. 3C). The liver of the SorOS group showed congested central vein, mild vacuolation of hepatocytes, and moderate Kupffer cells activity (Fig. 3D). The liver of the Amy + SorIP group showed congested central vein, congestion, and dilation of blood sinusoids in addition to Kupffer cells activity (Fig. 3E). The liver of the Amy + SorOS group showed mild congestion of the central vein, mild dilation of blood sinusoids, and a moderate increase of Kupffer cells activity (Fig. 3F).

On the other hand, livers of untreated EAC-bearing mice (EAC group) showed aggregations of pleomorphic, hyperchromatic, and darkly basophilic cells (these cells were assumed to be EAC cells) in the perivascular area around the dilated and congested central vein as well as a notable area of hepatocellular necrosis (Fig. 3G). The five treated groups showed notable improvement in liver structure with less damage as compared to the EAC group. In the EAC + Amy group, the liver showed a moderate number of pleomorphic cells with moderate degeneration (Fig. 3H). The liver of the EAC + SorIP group showed a small focal area of pleomorphic cells with moderate degenerative changes (Fig. 3I). The liver of the EAC + SorOS group showed a moderate focal area of pleomorphic cells and mild dilation of some blood sinusoids (Fig. 3J). The liver of the EAC + SorOS group showed the smallest focal area of the pleomorphic cells with the mildest congestion in some blood sinusoids (Fig. 3K). The liver of the EAC + SorOS group showed small diffuse pleomorphic cells with mild vacuolar degeneration of hepatocytes (Fig. 3L).

## Discussion

Mice injected with EAC cells and treated with Amy and/or Sor (IP or OS) showed a significant reduction in the ascitic fluid (tumor volume), the body weight, and viable tumor cell as compared to mice in the untreated EAC group. Among the five treated groups, the best antitumor effect (as revealed by less ascitic fluid, body weight, and viable EAC cells) was noticed in animals treated with both Amy and Sor with better effect for those injected IP by Sor. This antitumor effect could be attributed to enhanced phagocytes and cytokines production by Amy and/or Sor on EAC cells^18^. Our results also revealed that treatment with Amy and/or Sor not only significantly reduced the viable EAC cell count but also increased the count of non-viable cells. This infers that the antitumor action of Amy and Sor could be mediated through a direct inhibitory potential on the proliferation of tumor cells.

Hepatocytes are the main target for hepatic enzyme metabolism. Injury of these cells results in leakage of liver enzymes such as AST, ALT, and GGT from the liver into the circulation with an ultimate elevation of enzyme serum levels^19–21^. Hepatocytes are rigorously injured in animals with EAC cells^22^. This hepatic damage is accompanied by an increase in the levels of ALT and AST in the serum of EAC-bearing mice^22–24^. Consistent with these findings, we also found a significant elevation in ALT, AST, and GGT in the EAC group as compared to the control group. Reduced level of these hepatic damage enzymes in serum is associated with the antitumor potential of synthetic and natural anticancer compounds^19,25,26^. In agreement, we also found a significant reduction in the serum levels of these enzymes in EAC mice treated with Amy and/or Sor with best effect for animals treated with both Amy and SorIP. In line with our findings, another study reported that administration of Amy decreased the elevated ALT, AST, ALP, and GGT levels in *N*-nitrosodiethylamine-intoxicated rats^27^. Albumin and total proteins are other hepatic markers that are very important to follow up the progression of liver damage. We found a significant decrease in these two markers in EAC-bearing mice when compared to the control mice. Similarly, decreased levels in serum total protein and albumin were also observed in hepatic dysfunction cases^2,22^. Again, administration of Amy and/or Sor elevated these two markers in EAC-bearing mice with superior effect in co-treated mice, especially with Amy and SorIP. Similarly, Amy pretreatment increased albumin, total proteins in intoxicated rats^27^.

In the present study, treatment with Amy and/or Sor neutralized the effects induced by oxidative stress in EAC-bearing mice. This overall conclusion is based on our data which revealed that administration of Amy and/or Sor inhibited the lipid peroxides (MDA) and increased the antioxidant markers (GSH and SOD). In support, Amy increases the free radical scavenging activity through its stimulatory effect on GSH level and SOD activity in the liver of intoxicated rats^27^. The therapeutic potential of Amy is mainly attributed to its components that exhibit a wide range of biological effects including free radical scavenging^28^. Amy also improved the level of GSH in dimethylnitrosamine-induced liver fibrosis^27^. At a molecular level, mice treated with Amy and/or Sor showed a significant upregulation of the antioxidant-related *Nrf2* gene, with best effect in mice co-treated with Amy and SorIP, as compared to the untreated EAC-bearing mice. Nrf2 is mandatory for defending the body against cancer^29^.

It was found that tumor-induced angiogenesis is initiated by angiogenic cytokines such as basic fibroblast growth factor (bFGF) and VEGF that are expressed in the tumor itself^30^. The expression of VEGF is increased by the treatment of TGFβ, which is activated by MMP9^31^. Our data revealed a significantly upregulated expression of the migration-related *MMP9* gene and the angiogenesis-related *VEGF* gene in the liver of the untreated EAC-bearing mice. Our results are consistent with El Bakary, et al. (32) who reported a significant elevation in the expressions of *MMP9* and *VEGF* in EAC mice. This expression was downregulated following treatment with Amy and/or Sor, with best effect for Amy + SorIP.

Histopathological examination showed aggregations of pleomorphic, hyperchromatic, and darkly basophilic cells assumed to be EAC cells in the perivascular area around the dilated and congested central vein and hepatocellular necrosis in the liver of the EAC group. Additionally, degenerative changes such as loss of histo-architecture, due to microvascular fatty changes in addition to mild Kupffer cells activity were also observed. It was also reported that EAC cells can migrate from the peritoneal cavity and reach the liver causing liver injury^32^. EAC-bearing mice treated with Amy and/or Sor showed notable improvement in liver structure, especially in Amy + SorIP co-treated mice.

## Conclusions

Treatment of EAC-bearing mice with Amy and/or Sor reduced tumor burden and lipid peroxidation, improved antioxidant status, and restored the damaged hepatocytes. Amy and SorIP are more effective in ameliorating the effect of liver damage in EAC- bearing mice. Amy could improve the antitumor effect of Sor on EAC. Hence, it could be utilized as an adjuvant for Sor during cancer therapy. Therefore, further studies are encouraged to create a novel strategy targeting cancer cells using Amy and Sor co-therapy.

## Materials and methods

### Animals

Research ethical approval was obtained from the research ethical committee, Faculty of Science, Tanta University, Egypt, as established by the institutional animal care and use committee (IACUC). All methods were completed in accordance with ARRIVE guidelines. The experiment was carried out on female Swiss albino mice weighing 20–25 g and of 10–12 weeks ages. Mice were maintained under standardized conditions. Mice were kept in a controlled temperature environment with a 24 h cycle. All mice were adapted to the place for two weeks before the start of the experiment. The animals were provided with a normal diet and water ad libitum. Four EAC-bearing mice at day 14 of EAC intraperitoneal (IP) injection were obtained from the Cancer Biology Unit, Cairo, Al-Kaser Al-Eini, Egypt. In our lab, the ascitic fluid containing EAC cells was maintained and propagated by serial aseptic IP transplantation in mice. Each mouse was injected with 200 mL of 1 × 10^6^ EAC cells^33^. EAC cells filled the peritoneal cavity by fast division of cells, resulting in accumulation of ascitic fluid, and the animal could be died 17–18 days after EAC injection if did not receive appropriate treatment^3^.

### Experimental design

Seventy-two mice were divided into 12 groups (n = 6/group). Normal (control) group: mice were injected IP with normal saline (0.9% w/v, 300 µl/mouse). Amy group: mice were administrated amygdalin. SorIP group: mice were IP treated with sorafenib. SorOS group: mice were treated orally with sorafenib. Amy + SorIP group: mice were administered amygdalin and injected IP with sorafenib. Amy + SorOS group: mice were orally given amygdalin and sorafenib. EAC group: mice were injected IP by 200 mL of 1 × 10^6^ EAC cells and left for 14 days without treatment. EAC + Amy group: animals were injected once with EAC cells and 24 h later they were treated with amygdalin. EAC + SorIP: mice were inoculated with EAC cells and 24 h later they were IP injected with sorafenib. EAC + SorOS: mice were inoculated once with EAC cells and 24 h later they were orally administered sorafenib. EAC + Amy + SorIP: mice were inoculated once with EAC and 24 h later they were co-treated with amygdalin and sorafenib (IP). EAC + Amy + SorOS group: mice were inoculated with EAC and 24 h later they were co-treated with amygdalin and sorafenib. All treatments were given daily for 14 days and the doses of amygdalin (300 mg/kg mouse) and sorafenib (30 mg/kg mouse for both IP and OS) were chosen based on a pilot study with aid of previous studies^34,35^. Amygdalin (Vitamin B17) and sorafenib were purchased from Sigma-Aldrich and BAYER companies, respectively. The animals were weighed at the beginning of the experiment (initial body weight, g), at the end of the experiment (final weight, g) and the mean body weight change (g) was then calculated by subtracting the initial weight from the final weight. It is well-known that changes in body weight of EAC-bearing mice is an additional indirect measure of changes in tumor mass in these animals.

### Sampling

At the end of the experimental period (2 weeks), overnight fasted rats have sacrificed for 24 h. After the last treatment, blood samples were centrifuged in clean glass tubes for 15 min at 3000*×g* to get clear, non-hemolyzed sera. Eppendorf tubes with labels were immediately shipped to − 20 °C; the sera were frozen for biochemical analysis. After euthanization by exsanguination, livers were immediately removed and some specimens were fixed in 10% formalin (pathological investigation), and the others were either homogenized (biochemical assay) or frozen in − 70 °C (RNA extraction).

### Tumor (ascitic fluid) volume and EAC count

At the end of the experiment (day 14), ascitic fluid containing EAC cells was withdrawn from the peritoneal cavity of each mouse before they were dissected. A graduated centrifuge tube was used to measure how much ascitic fluid was collected. Following the suspension of EAC cells in sterile isotonic saline, a Neubauer hemocytometer was used to count the total, viable and non-viable EAC cells^33^.

### Assessment of biochemical parameters

The serum level of liver damage enzymes [aspartate transaminase (AST), alanine transaminase (ALT), gamma-glutamyl transferase (GGT)], albumin, and total proteins were measured using commercially available kits (Biomed Diagnostics, Cairo, Egypt). Liver homogenates were prepared as previously described^36^. The hepatic levels of lipid peroxidation marker malondialdehyde (MDA) and the antioxidant markers reduced glutathione (GSH) and superoxide dismutase (SOD) were measured colorimetrically using kits purchased from Biodiagnostics and as previously described^37,38^.

### Real-time PCR

Real-time PCR was used to determine changes in the relative expression of *Nrf2*, *MMP9*, and *VEGF* genes in liver specimens of all groups. Total RNA was first isolated and then reverse transcribed into cDNA by kits purchased from Thermo Scientific, USA (# K0731 and #EP0451, respectively). The sequences of primers were as following: F: 5′ CACATCCAGACAGACACCAGT 3′ and R: 5′ CTACAAATGGGAATGTCTCTG C 3′ for *Nrf2*; F: 5′ TCGAAGGCGACCTCAAGTG 3′ and R: 5′ TTCGGTGTAGCTTTG GATCCA 3′ for *MMP9*; F: 5′ GATCATGCGGATCAAACCTCACC 3′ and R: 5′ CCTCCGGACCCAAAGTGCTC 3′ for *VEGF*; F: 5′ CATGGATGACGATATCGCT 3′ and R: 5′ CATGAGGTAGTCTGTCAGGT 3′ for *β* *actin* (internal control). Thermal and melting curve conditions were done as previously detailed^39–41^. The fold change in gene expression was determined using the 2^−∆∆Ct^ method.

### Histopathological investigation

Liver tissues were dehydrated in ascending series of ethanol, cleared in xylene, embedded in paraffin wax, sectioned at 5 µm thickness by a microtome, stained with eosin and hematoxylin, examined, and photographed under a light microscope to detect histopathological changes.

### Statistical analysis

GraphPad Prism 5.0 was used to analyze the data. The experimental results were expressed as mean ± standard error mean (SEM). Data were assessed by one-way analysis of variance (ANOVA) followed by the Tukey test for multiple comparisons test. Values for which P < 0.05 were considered statistically significant.

### Institutional review board statement

The study was conducted according to the guidelines of ARRIVE and approved by Ethical committee at the Faculty of Science, Tanta University.

## Data Availability

The data presented in this study are available on request from the corresponding author.
